# Laparoscopic High Uterosacral Ligament Suspension vs. Laparoscopic Sacral Colpopexy for Pelvic Organ Prolapse: A Case-Control Study

**DOI:** 10.3389/fmed.2022.853694

**Published:** 2022-03-04

**Authors:** Giuseppe Campagna, Lorenzo Vacca, Giovanni Panico, Giuseppe Vizzielli, Daniela Caramazza, Riccardo Zaccoletti, Monia Marturano, Roberta Granese, Martina Arcieri, Stefano Cianci, Giovanni Scambia, Alfredo Ercoli

**Affiliations:** ^1^Department of Woman, Child, and Public Health, Fondazione Policlinico Universitario A. Gemelli Istituto di Ricovero e Cura a Carattere Scientifico (IRCCS), Rome, Italy; ^2^Department of Medicinal Area (DAME) Clinic of Obstetrics and Gynecology, Santa Maria della Misericordia Hospital, Azienda Sanitaria Universitaria Friuli Centrale, Udine, Italy; ^3^Obstetrics and Gynecology Unit, P. Pederzoli Hospital, Peschiera del Garda, Verona, Italy; ^4^Department of Biomedical, Dental, Morphological and Functional Imaging Science, University of Messina, Messina, Italy; ^5^Department of Human Pathology in Adult and Childhood “G. Barresi, ” University of Messina, Messina, Italy; ^6^Department of Woman, Child and Public Health, Catholic University of Sacred Heart, Rome, Italy

**Keywords:** laparoscopic high uterosacral ligament suspension, laparoscopic sacral colpopexy, pelvic organ prolapse, laparoscopic surgery, urogynecology

## Abstract

**Introduction:**

Laparoscopic sacral colpopexy is the gold standard technique for apical prolapse correction but it is a technically challenging procedure with rare but severe morbidity. Laparoscopic high uterosacral ligament suspension could be a valid technically easier alternative using native tissue.

**Material and Methods:**

In the period from 2015 to 2018, 600 women were submitted to laparoscopic sacral colpopexy while 150 to laparoscopic high uterosacral ligament suspension in three Italian urogynecology referral centers. We enrolled women with apical prolapse stage ≥2 alone or multicompartment descensus. To reduce allocation bias, we performed a propensity matched analysis. Women undergoing laparoscopic high uterosacral ligament suspension surgery were matched 1:2 to women undergoing laparoscopic sacral colpopexy. The cumulative proportion of relapse-free women in time was analyzed by the Kaplan–Meier method. The primary objective of this multicenter case-control retrospective study was to compare the recurrence rate while the secondary objectives were to compare feasibility, safety, and efficacy of laparoscopic sacral colpopexy and laparoscopic high uterosacral ligament suspension in surgical treatment of pelvic organ prolapse.

**Results:**

Three hundred and nine women were enrolled (103 laparoscopic high uterosacral ligament suspension; 206 laparoscopic sacral colpopexy). Median operatory time was significantly shorter in the laparoscopic high uterosacral ligament suspension group (*P* = 0.0001). No statistically significative difference was found in terms of estimated blood loss, admission time, intraoperative, and major early postoperative complications, postoperative pelvic pain, dyspareunia and *de novo* stress urinary incontinence. Surgical approach was the only independent risk factor for prolapse recurrence (RR = 6.013 [2.965–12.193], *P* = 0.0001). The objective cure rate was higher in the laparoscopic sacral colpopexy group (93.7 vs. 68%, 193/206 vs. 70/103, *P* = 0.0001) with a highly reduced risk of recurrence (RR = 5.430 [1.660–17.765]). Median follow up was 22 months.

**Conclusion:**

Both techniques are safe, feasible, and effective. Laparoscopic sacral colpopexy remains the best choice in treatment of multicompartment and advanced pelvic organ prolapse while laparoscopic high uterosacral ligament suspension could be appropriate for moderate and isolated apical prolapse when laparoscopic sacral colpopexy is not suitable for the patient or to prevent prolapse in women at high risk at the time of the hysterectomy.

## Introduction

Pelvic organ prolapse (POP) is a common female condition which involves the descent alone or in combination of the bladder, the rectum, the uterus (cervix) or the apex of the vagina (in case of previous hysterectomy) from their normal position in the pelvis with a consequent bulge into the vagina (1, 2). Although rarely resulting in severe morbidity or mortality, POP with is lower genital, urinary, and gastrointestinal tracts symptoms, affects the quality of life up to 40% of all women influencing daily activities, sexual function, and exercise (3). Its presence can have a negative impact on body image and sexuality (4, 5). Both the incidence and prevalence of POP surgery tend to increase with age. The estimated incidence of POP surgery ranges from 1.5 to 1.8 per 1,000 women year with the incidence peaking in women between 60 and 69 years (6).

Even though the vaginal approach continues to be the most common contributing up to 90% of surgical intervention (7), the know high rate of POP recurrence after transvaginal surgery with native tissue and the increasingly frequent reports on mesh-related complications with the consequent FDA transvaginal mesh-related litigation, have caused a decrease in the practice of this type of surgery in favor of laparoscopic abdominal procedures (8–10).

Nowadays, laparoscopic sacral colpopexy (LSCP) can be considered the gold standard technique for apical prolapse correction because of its lower recurrence and reoperation rates than a variety of vaginal procedures (vaginal sacrospinous colpopexy, uterosacral colpopexy, and transvaginal mesh) with a longer operating time as the only disadvantage (7). However, LSCP is a technically challenging procedure, because of the need of deep pelvic dissections and high skill in suturing and it is associated with rare but severe morbidity, with documented cases of vascular injuries and sacral nerve roots damage and consequent chronic constipation and pain (11, 12). For these reasons, new strategies were investigated to suspend vaginal apex in a technically easier way avoiding the most difficult and dangerous steps of LSCP.

Laparoscopic high uterosacral ligament suspension (L-HUSLS) is an alternative surgical intervention for apical prolapse correction using native tissue with feasibility, safety, and efficacy already demonstrated by several studies (13–18). When compared with the conventional vaginal approach, the laparoscopic procedure has similar objective success rates and a small number of ureteral injuries (13–16).

Despite the current state of affairs, the previously published studies able to compare the two techniques are few and characterized by a small sample of size (13, 19). For these reasons, our case-control study aimed to compare feasibility, safety, efficacy, and prolapse recurrence rates of LSCP and L-HUSLS in a high-volume urogynecology practice.

## Materials and Methods

**Figure 1:**
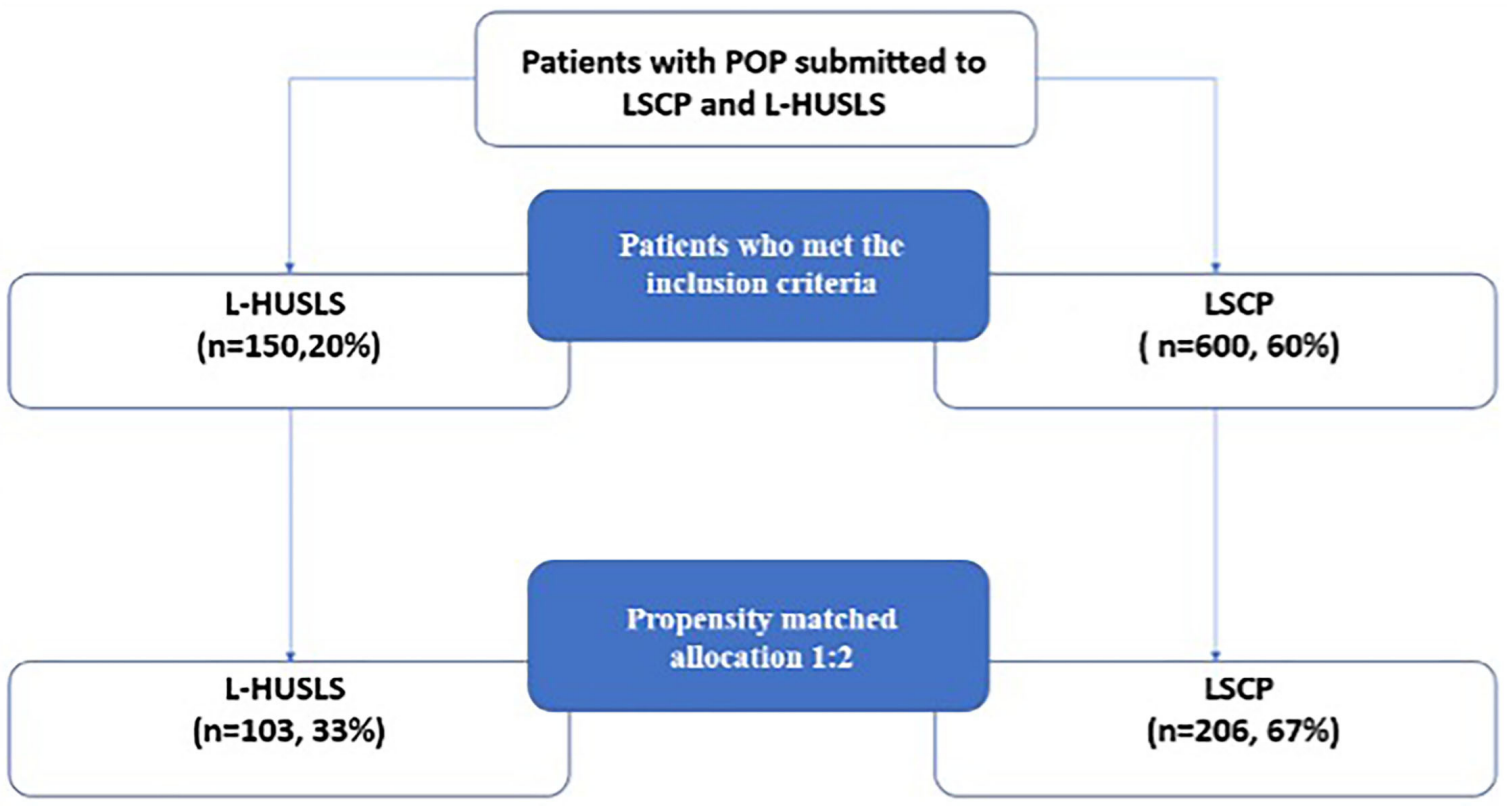
Study design and selection process. In the period from 2015 to 2018, patients in the Control group and 150 patients in the Case Group were enrolled. Because of the nonrandomized nature of the study design and the possible allocation biases arising from the retrospective comparison between groups, we performed a propensity matched analysis. LSCP, laparoscopic sacral colpopexy; L-HUSLS, laparoscopic high uterosacral ligament suspension.

This is a multicenter retrospective case control study including patients with apical POP (ICS) stage ≥ 2 (1) alone or in association with anterior and/or posterior descensus who underwent to L-HUSLS (Cases) and LSCP (Controls). The study was conducted at urogynecology referral centers of Fondazione Policlinico Universitario A. Gemelli IRCCS of Rome, Azienda Ospedaliera Universitaria Gaetano Martino of Messina and Clinica Polispecialistica Convenzionata Pederzoli of Peschiera d/G. In the period from 2015 to 2018, we enrolled 600 patients in the Control group and 150 patients in the Case Group (Figure 1). Both types of surgical techniques were performed in all the hospitals involved in the study.

### Inclusion and Exclusion Criteria

Inclusion criteria were the following: postmenopausal patients with POP (ICS) stage ≥ 2 for the apical compartment; age <80 years; no uterine cervix dysplasia or endometrial disorders; no uterine size larger than conform 12 weeks' gestation; no previous longitudinal major abdominal surgery.

We excluded patients with anesthesiologic contraindications for minimally invasive approach. Cases were retrieved from our institutional database.

Four expert uro-gynecological surgeons (GC, GP, AE, and RZ) with a minimum of 30 LSCP and 30 L-HUSLS per year, prior to this study, performed all procedures.

The surgical selection was based on prolapse type and grade, surgeon preference, risk factors, and women history of previous surgery and preference. Additional procedures performed when indicated include total or supracervical hysterectomy, anterior colporrhaphy, and suburethral sling.

All patients received an upfront explanation of the surgical approach. Women signed written consent to undergo the described procedure and to permit data use.

The study was approved by the three hospitals institutional review boards and has been carried out in accordance with The Code of Ethics of the World Medical Association (Declaration of Helsinki) for experiments involving humans.

### Preoperative and Perioperative Assessment

Preoperative assessment involving exhaustive history, physical examination, urodynamic testing, smear test and ultrasound scan was performed by an urogynecologist of each surgical team. When urogenital or ano-rectal malignant pathologies were suspected, supplementary exams and/or imaging were executed. POP was classified according to the Pelvic organ prolapse quantification (POP-Q) system published by the International Continence Society (1). Preoperatively women were questioned about urinary, bowel, and sexual function.

### Surgical Technique

The three surgical teams performed all procedures using a standard technique in accordance with what we previously published (20–23).

In case of LSCP two adequately shaped polypropylene type 1 mesh fixed with non-absorbable sutures were used to correct the POP. Finally, the anterior mesh was fixed to the longitudinal vertebral ligament at L5-S1 level with 1–0 non absorbable suture on a noncutting needle.

When L-HUSLS was performed two polydioxanone 1 suture stitch (PDS^®^ Ethicon, Somerville, NJ, USA) were used to suspend vaginal apex. During the procedure, we mobilized and lateralized the ureters and hypogastric nerves to avoid injuries.

### Follow-Up

We used Clavien–Dindo's (CD Grade) classification for grading postoperative complications during the first 30 days after surgery (24) and the ICS/IUGA joint report on the terminology for pelvic floor dysfunction (25) to describe surgical results.

We considered as an anatomic surgical failure a POP stage ≥ 2 in any compartment.

Patients underwent postoperative routine follow up at 1, 3, 6, and 12 months after the intervention and then yearly which were performed by an urogynecologist of each group. Urodynamic testing was repeated 12 months after the surgical treatment in all women without problems.

The Patient Global Impression of Improvement (PGI-I) questionnaire administered at 3 and 12 months (26) was used to evaluate the overall postoperative patient satisfaction. Women were asked about the changing of urinary and/or bowel and/or sexual function after the surgical procedure. During medical interview, sexually active patients were asked if they were affected by dyspareunia, defined as a perceived pain or discomfort during sexual intercourse.

### Statistical Analysis

Because of the non-randomized nature of the study design and the possible allocation biases arising from the retrospective comparison between groups, we performed a propensity matched analysis (Figure 1). Propensity-matched comparison attempts to estimate the effect of a treatment by accounting for possible factors (e.g., constitutional variables) that predict receiving the treatment. Propensity-matched comparison aims to reduce biases arising from different covariates (27–29). A propensity score was developed through a multivariable logistic regression model. Age, body mass index, the preoperative stage of apical prolapse, were included in the model. Patients undergoing L-HUSLS surgery were matched 1:2 to patients undergoing LSCP using a caliper width ≤ 0.1 standard deviations of the logit odds of the estimated propensity score. Univariate analysis was performed to verify any difference between the two groups. The χ^2^ analysis or Fisher's exact test were used, when appropriate, for categorical variables and the Student *t*-test and Mann–Whitney test, when appropriate, for continuous variables. Differences between the groups were considered statistically significant at *p* < 0.05 (95% confidence interval). The Kaplan–Meier method was used to analyze the cumulative proportion of relapse-free patients in time. The NCSS statistical software program, version 11.0 (NCSS Statistical Software, Kaysville, UT), was used.

## Results

After propensity matching, 103 patients were in the case cohort and 206 patients were in the control cohort. Patient characteristics of the two cohorts are shown in Table 1. No difference between groups was found in terms of age, BMI, comorbidities, previous POP surgery, parity, prior hysterectomy. Smokers were prevalent in the control group.

**Table 1:** Baseline patients characteristics.

**Variables**	**L-HUSLS^+^** **(*N*) (%)**	**LSCP^a^** **(*N*) (%)**	***p*-value**
**All cases**	103	206	–
**Age**			
<65 years	74 (71.8)	147 (71.4)	0.929
≥65 years	29 (28.2)	59 (28.6)	
**Body mass index (Kg/m** ^**2**^ **)**			
<25	44 (42.7)	94 (45.6)	0.716
≥25	59 (57.3)	112 (54.4)	
**Diabetes**			
Yes	7 (6.8)	13 (6.3)	0.870
No	96 (93.2)	193 (93.7)	
**COPD^◦^**			
Yes	4 (3.9)	4 (1.9)	0.448
No	99 (96.1)	202 (98.1)	
**Parity**			
Yes	102 (99.0)	199 (96.6)	0.277
No	1 (1.0)	7 (3.4)	
Prior POP^*^surgery			
Yes	7 (6.8)	25 (12.1)	0.169
No	96 (93.2)	181 (87.9)	
**Prior hysterectomy**			
Yes	11 (10.7)	40 (19.4)	0.053
No	92 (89.3)	166 (80.6)	
**Smoking**			
Yes	5 (4.9)	28 (13.6)	**0.01**
No	98 (95.1)	178 (86.4)	
Preoperative SUI^**^			
Yes	25 (24.3)	55 (26.7)	0.681
No	78 (75.7)	151 (73.3)	
**POP Q stage anterior**			
1–2	40 (38.8)	35 (17.0)	**0.0001**
3–4	63 (61.2)	171 (83.0)	
**POP Q stage apical**			
2	48 (46.6)	91 (44.2)	0.717
3–4	55 (53.4)	115 (55.8)	
**POP Q stage posterior**			
1–2	103 (100)	187 (90.8)	**0.001**
3–4	0	19 (9.2)	

+ *Laparoscopic sacral colpopexy.*

a *Laparoscopic high uterosacral ligament suspension (L-HUSLS).*

◦ *Chronic obstructive pulmonary disease.*

* *Pelvic organ prolapse.*

** *Stress urinary incontinence.*

*LSCPa, aLaparoscopic sacral colpopexy; L-HUSLS+, +Laparoscopic high uterosacral ligament suspension*.

Even though the distribution of apical POP Q stage was similar among two groups there was a trend toward more severe (stage III/IV) anterior and posterior prolapse in the sacral colpopexy cohort (83.0 vs. 61.2% *p* = 0.0001 for the anterior descensus and 0 vs. 9% for the posterior one) Perioperative parameters are summarized in Table 2.

**Table 2:** Perioperative data.

**Variables**	**L-HUSLS** **(*N*) (%)**	**LSCP** **(*N*) (%)**	***p*-value**
**All cases**	103	206	–
**Operative time (minimum) (median) (range)**	120 (60–270)	190 (110–290)	**0.0001**
**Estimated blood loss (mL) (median) (range)**	70 (0–130)	50 (0–110)	0.965
**Concomitant procedures**			
Anterior colporrhaphy	66 (64.1)	0	**0.0001**
Sub-urethral sling	12 (11.7)	1 (0.5)	**0.0001**
**Intraoperative complications**	–	1 (0.5)	0.479
Early (<30 days) major postoperative complications^*^	–	–	n.a.^**^
**Hospital stay (days) (median) (range)**	2 (1–4)	2 (1–4)	0.186

* *≥3 according to Clavien-Dindo scale (xx);*

** *n.a., not applicable*.

Median OT was significantly shorter in the L-HUSLS group 120 min vs. 190 min (*p* = 0.0001). No statistically significative difference was found in terms of estimated blood loss, admission time, intraoperative complication and major early postoperative complication. We registered 1 (0.5%) intraoperative complication: a bladder injury in LSCP group.

All women with uterus underwent total hysterectomy in the case group and subtotal hysterectomy in the control group.

There were no significant differences between the two cohorts in terms of postoperative pelvic pain (1% in the case group vs. 2.4% in the control group, *p* = 0.382), dyspareunia (2% in the case group vs. 6% in the control group, *p* = 0.612) and *de novo* stress urinary incontinence (6.8% in the case group vs. 13.1% in the control group, *p* = 0.123).

There was one case of LSCP mesh erosion (0.5%) managed conservatively with vaginal estrogen. There were four cases of urinary retention in the L-HUSLS group which all resolved spontaneously within 1 week, two cases of urinary infection in the LSCP group treated successfully with antibiotics and 1 case of Deep vein thrombosis cured with anticoagulant therapy.

PGI-I score for both groups is summarized in Table 3.

**Table 3:** Patient global Impression of Improvement (PGI-I).

	**Very much better**	**A little better**
	**Much better**	**No change**
	**Score 1–2**	**Score 3–4**
**L-HUSLS (*N*) (%)**	74 (72%)	29 (28%)
**LSCP (*N*) (%)**	194 (94%)	12 (6%)
***p*** **-value**	**0.0001**	**0.0001**

In the univariate and multivariate analysis surgical approach was the only independent risk factor for POP recurrence (RR = 6.013; CI: 2.965–12.193, *p* = 0.0001; Table 4).

**Table 4:** Risk factors for prolapse recurrence.

**Variable**	**Univariate analysis**		Multivariate analysis^*^	
	**Risk ratio (95% CI)**	*p*-value^**^	**Risk ratio (95% CI)**	*p*-value^**^
**Age**				
≤ 65^§^	0.497 (0.222–1.116)	0.107	2.110 (0.897–4.964)	0.087
>65				
**Body mass index (BMI)**				
≤ 25 Kg/m^2^	1.123 (0.593–2.128)	0.748	–	–
>25 Kg/m^2§^				
**Surgical approach**				
L-HUSLS^§^	6.691 (3.324–13.470)	**0.0001**	6.013 (2.965–12.193)	**0.0001**
LSCP				
**COPD**
Yes§	0.850 (0.811–0.892)	0.237	–	–
No				
**Prior POP surgery**				
Yes^§^	0.363 (0.084–1.574)	0.159	1.586 (0.336–7.491)	0.560
No				
**Prior hysterectomy**				
Yes^§^	0.921 (0.386–2.196)	0.853	–	–
No				
**POP Q stage anterior**				
1–2	1.333 (0.610–2.914)	0.574	–	–
3–4§				
**POP Q stage apical**				
2	0.924 (0.490 – 1.741)	0.872	–	–
3–4§				
**POP Q stage posterior**				
1–2	0.845 (0.804–0.888)	0.063	1.198 (0.01–8.493)	0.998
3–4§				

* *Multivariate analysis with method backward stepwise was performed for variable with p < 0.2 at univariate analysis.*

** *Bold cases are statistically significant: p < 0.05*.

Anatomic outcomes are presented in Table 5. The objective cure rate was higher in the LSCP group (93.7 vs. 68%, *p* = 0.0001) with a highly reduced risk of prolapse recurrence (RR = 5.430, CI: 1.660–17.765). The length of follow-up was similar with a median of 22 months in both groups.

**Table 5:** Pattern of recurrent prolapse according to surgical approach.

**Variable**	***N* (%)**	**L-HUSLS (%)**	**LSCP (%)**	***p*-value**	**Risk ratio** **(95% CI)**
**All cases**	309	103	206	–	–
**Recurrences**	46 (14.9)	33 (32)	13 (6.3)	0.0001	5.430 (1.660–17.765)
Anterior	26 (56.5)	18 (54.5)	8 (61.6)	0.0001	5.743 (2.768–11.917)
Apical	4 (8.7)	4 (12.1)	0	0.0001	7.944 (2.544–24. 809)
Posterior	2 (4.3)	1 (3.0)	1 (7.7)	0.011	6.309 (1.251–31.832)
Multicompartmental	14 (30.5)	10 (30.4)	4 (30.7)	0.002	5.430 (1.660–17.765)

**Figure 2:**
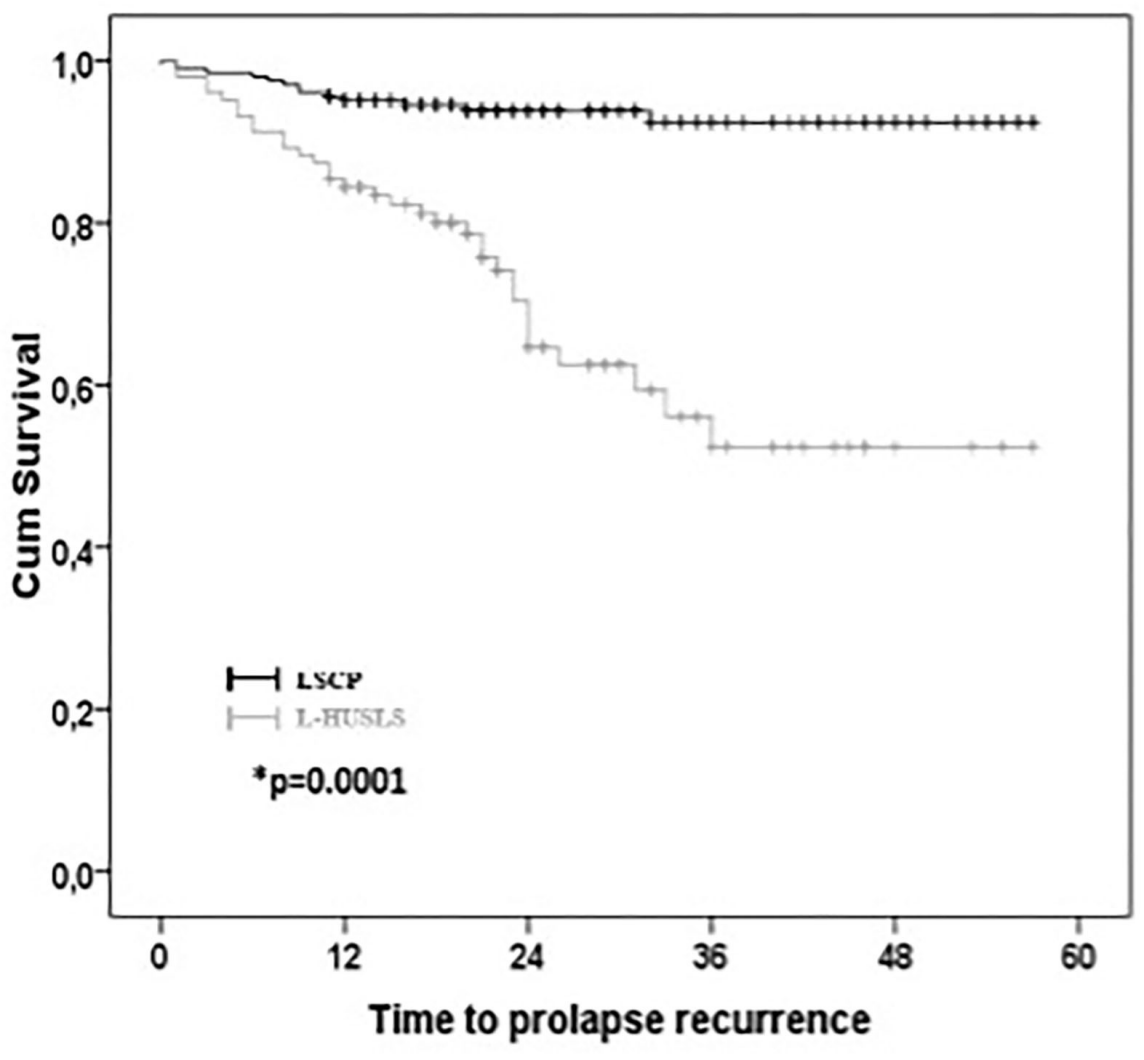
Kaplan–Meier survival curves for anatomical recurrence. Kaplan–Meier curves of objective recurrence in the whole population.

Kaplan–Meier curves of objective recurrence in the whole population are shown in Figure 2.

## Discussion

Our study demonstrates that both laparoscopic procedures are safe with rare and minor perioperative complications and a superimposable admission time in accordance with the main principles of the mini-invasive approach. In addition, L-HUSLS showed significative lower OT than LSCP. The absence of ureteral injuries further emphasizes the safety of the L-HUSLS techniques if compared to the 11% of the ureteral damaging rate described in previous studies on V-USLS (30). One of the perceived benefits of the laparoscopic approach includes the magnified view of the operative field allowing an easier dissection, a more precise suture placement and a better visualization of vital structures. This may help to reduce the possible damage to the ureter which could be further protected by a prophylactic ureterolysis before the apical suspension. This confirms what already published studied demonstrated about the minimal ureteric injury rates during this laparoscopic procedure (14, 15, 31, 32). Mesh erosion rate in LSCP group with only in 1 case (0.5%) was lower compared to data already reported in literature (7, 12, 33, 34). This may be related to the surgical technique, to the prothesis material (polypropylene type 1 with mesh weight ranging from 16 to 65 g/m^2^) (35) and to time of follow up. The use of a standard subtotal hysterectomy avoids the communication between the vaginal and the abdominal cavities and the consequent exposition of the surgical bed to the vaginal microbiota. This element, combined with the devascularization of the vaginal cuff caused by uterus removal, may play a significative role in the evolution of subsequent erosion. Even though the length of the follow up in the study is enough to detect mesh related complications, it's still too short to compare our results with those included in the study by Nygaard et al. (33) which report a rate of mesh erosion of 10.5% at 7 years. The incidence of *de novo* SUI was higher in the LSCP group but not in a significative manner. Our data don't differ from those already published in literature (13, 36).

The present study founded that LSCP has higher objective success rate than L-HUSLS in multicompartment advanced pelvic organ prolapse. Regarding LSCP our anatomic outcome was similar to those already described in literature (33, 34). The recurrence rate in patients underwent L-HUSLS were higher than those previously published (13, 15, 37, 38). This may be related to the larger sample size investigated, the longer follow up time and the highest grade of preoperative pelvic organ prolapse. Filmar et al. (13) reported an anatomic success rate for L-HUSLS of 89.7 % in only 29 patients with a preoperative POP stage 2 and a follow up of 6 months. Haj Yahya et al. reported with the same procedure an anatomic success rate of 91.3% but 54% of the population had a preoperative apical prolapse of grade I, and 22.9% of grade II with a FUP of 17.5 months (38). The PGI-I reflects the anatomical outcome with a significative higher percentage of women in the LSCP group with a score of 1–2.

To better evaluate the effectiveness of the two techniques, the univariate and multivariate analysis was performed to understand which could be possible confounding risk factors for surgical failure. However, none of the tested independent variables, including preoperative POP Q stage, had an influence on anatomic recurrence except for the type of surgical procedures. This may be related to the characteristics of our population characterized by multicompartmental POP. L-HUSLS is a fascial technique indicated for the correction of apical prolapse. Higher grade of apical prolapse is often associated with anterior or posterior descensus. While LSCP is often able to correct the defects in all the compartments in this clinical situation, L-HUSLS often requires an additional vaginal native tissue repair. This exposes the women to the augmented risk of surgical failure of fascial surgery. Significantly we observed that 85% of prolapse recurrence in L-HUSLS group involved the anterior compartment even though the procedure was associated with anterior colporrhaphy in 66 cases (64.1 %). The anatomical failure rate in the anterior compartment (28/103, 27%) is similar to those described by Maher's Cochrane indicating that 27–42% of women would have a recurrence after native tissue repair (39). Taking in consideration only the apical compartment we observed that, even if the LSCP remains the referral treatment with an objective success rate of 98% (202/206), the anatomical success rate for the L-HUSLS technique increases up to 87% (89/103). This demonstrates that although L-HUSLS is significantly less effective than LSCP in advanced multi- component prolapses, it remains an effective treatment for isolated and mild apical prolapses bypassing the limits of the vaginal routes (such as chronic pelvic pain, dyspareunia, ureteral obstruction) (14, 15). There are particular clinical situations such as the need for a total hysterectomy (in case of cervical pathology) and the presence of contraindications to the positioning of the prosthetic material (patients at high risk of mesh infection) in which the L-HUSLS may play a significative role. Moreover, results showed a significative shorter operative time of L-HUSLS. This would be an advantage for patients who, due to their comorbidities, cannot sustain a long surgical procedure. Thanks to its demonstrated safety and feasibility this technique should take in consideration for POP prevention in patients undergoing total hysterectomy for benign indication in which risk factors for future descensus have been recognized.

This was a pilot study before planning a multicentric prospective study with a larger sample. In the absence of specific questionnaires, women completed the PGI-I questionnaire and expressed their satisfaction with the surgical treatment in terms of sexual function and bulge symptom resolution. In our prospective study, we plan to evaluate additional subjective outcomes.

Strengths of our study include large sample size, the multicentric setting in high volume hospitals, all participating surgeon performing both techniques and the long follow up.

Limitations of our study include those inherent to cohort studies. Because of the possibility of selection bias due to the absence of randomization we balanced the differences in patient characteristics between groups by using propensity score-matching. Although differences remained between the matched groups related to the preoperative POP Q stage anterior and posterior, we have attempted to address this discrepancy using the regression modeling.

## Conclusion

In conclusion, both presented techniques suggest safety, feasible, and efficacy in the treatment POP. LSCP still remains (remove the extra space) to be the best choice in the treatment of multicompartment and advanced pelvic organ prolapse while L-HUSLS appears to be well-appropriate for moderate and isolated apical prolapse when LSCP is not suitable for the patient or to prevent prolapse in patients at high risk at the time of the hysterectomy.

## Data Availability Statement

The raw data supporting the conclusions of this article will be made available by the authors, without undue reservation.

## Ethics Statement

The studies involving human participants were reviewed and approved by Comitato Etico Policlinico Universitario A. Gemelli—Largo Agostino Gemelli 8, 00168 Roma—Prot. ID 3487. The patients/participants provided their written informed consent to participate in this study.

## Author Contributions

GC, AE, LV, and RZ: conception and design. LV, RZ, and MM: acquisition of data. GV, SC, and GP: analysis and interpretation of data. LV, GP, MA, and DC: drafting of the manuscript. AE, GC, RG, and GS: critical revision of the manuscript. All authors contributed to the article and approved the submitted version.

## Conflict of Interest

The authors declare that the research was conducted in the absence of any commercial or financial relationships that could be construed as a potential conflict of interest.

## Publisher's Note

All claims expressed in this article are solely those of the authors and do not necessarily represent those of their affiliated organizations, or those of the publisher, the editors and the reviewers. Any product that may be evaluated in this article, or claim that may be made by its manufacturer, is not guaranteed or endorsed by the publisher.
