# Supplementary material for: Laparoscopic High Uterosacral Ligament Suspension vs. Laparoscopic Sacral Colpopexy for Pelvic Organ Prolapse: A Case-Control Study
Source: Front Med (Lausanne). 2022 Mar 4;9:853694. doi: 10.3389/fmed.2022.853694 (PMC8930828; doi:10.3389/fmed.2022.853694)
Supplement: Supplementary file 1 [file Table_1.DOCX]

# Supplemental Table 1. Postoperative complications details

| **Variables** | **L-HUSLS**  **(N)(%)** | **LSCP**  **(N)(%)** | **p-value** |
| --- | --- | --- | --- |
| **All cases** | 103 | 206 | - |
| **Conversion to laparotomy** | 0 | 0 | n.a.* |
| **Re-intervention** | 0 | 0 | n.a.* |
| **Pelvic Pain** | 1 (1.0) | 5 (2.4) | 0.382 |
| **Dyspareunia** | 2 (1.9) | 6 (2.9) | 0.612 |
| **Ureteral obstruction** | 0 | 0 | n.a.* |
| **De novo SUI** | 7 (6.8) | 27 (13.1) | 0.123 |
| **Mesh erosion** | 0 | 1 (0.5) | 0.479 |
| **L5-S1 discitis** | 0 | 0 | n.a.* |
| **Hematoma needing drainage** | 0 | 0 | n.a.* |
| **Urinary retention** | 4 (3.9) | 0 | **0.004** |
| **Urinary infection** | 0 | 2 (1.0) | 0.316 |
| **Deep vein thrombosis** | 0 | 1 (0.5) | 0.479 |

*n.a.: not applicable**.**
